# Feasibility and outcome of third-generation transcatheter aortic valve implantation in patients with extra-large aortic annulus

**DOI:** 10.1007/s00392-023-02278-1

**Published:** 2023-07-31

**Authors:** Alexander Hof, Hendrik Wienemann, Verena Veulemans, Sebastian Ludwig, Tanja Katharina Rudolph, Simon Geißen, Elmar Kuhn, Kaveh Eghbalzadeh, Sabine Bleiziffer, Tobias Zeus, Alexander Sedaghat, Niklas Schofer, Stephan Baldus, Matti Adam, Victor Mauri

**Affiliations:** 1 Department of Cardiology, Faculty of Medicine, University Hospital Cologne, Clinic III for Internal Medicine, University of Cologne, Kerpener Str. 62, 50937 Cologne, Germany; 2 Division of Cardiology, Pneumology, and Vascular Medicine, Department of Medicine, University Hospital Duesseldorf, Duesseldorf, Germany; 3 Department of Cardiology, University Heart and Vascular Center Hamburg, Hamburg, Germany; 4 Clinic for General and Interventional Cardiology/Angiology, Heart and Diabetes Center North Rhine-Westphalia, Bad Oeynhausen, Germany; 5 Heart Center, Department of Cardiothoracic Surgery, University Hospital of Cologne, Cologne, Germany; 6 Department of Cardiothoracic Surgery, Heart and Diabetes Center North Rhine-Westphalia, Bad Oeynhausen, Germany; 7 Heart Center Bonn, Department of Medicine II, University Hospital Bonn, Bonn, Germany

**Keywords:** Transcatheter aortic valve implantation, TAVI, Aortic annulus, Annulus anatomy, Aortic valve stenosis

## Abstract

**Background:**

Many patients in need for transcatheter aortic valve implantation (TAVI) present with an aortic annulus size larger than recommended by the manufacturer’s instructions for use (IFU).

**Aims:**

To investigate procedural and short-term safety and efficacy of TAVI in patients with extra-large annuli (ELA).

**Methods:**

30-day clinical outcome and valve performance as defined by VARC 3 of 144 patients with an aortic annulus size exceeding the permitted range were compared to a propensity-score matched control cohort of patients with an aortic annulus size consistent with the IFU.

**Results:**

Area and perimeter was 730.4 ± 53.9 mm^2^ and 96.7 ± 6.5 mm in the ELA group. Technical (96.5% vs. 94.4%) and device success (82.3% vs. 84.5%) were comparable in patients with ELA (annulus area 730.4 ± 53.9 mm^2^) and matched controls (annulus area 586.0 ± 48.2 mm^2^). There was no significant difference in 30-day mortality rate, major intraprocedural complications, type 3 or 4 bleedings, major vascular complications, or stroke. Moderate paravalvular leakage (PVL) occurred more frequent in the ELA group (8.9% vs 2.2%; *p* = 0.02). The rate of new pacemaker implantation was 7.0% in the ELA cohort and 15.0% in the control cohort, respectively (*p* = 0.05).

**Conclusion:**

Treatment of ELA patients with third-generation TAVI prostheses is feasible and safe, providing similar device success and complication rates as in matched controls with regular-sized aortic annulus. Post-interventional pacemaker implantation rates were low compared to the control group, yet incidence of moderate PVL remains problematic in ELA patients.

**Supplementary Information:**

The online version contains supplementary material available at 10.1007/s00392-023-02278-1.

## Introduction

Severe aortic stenosis (AS) is a common condition in the elderly population, being associated with a poor prognosis if left untreated [1] [2] [3]. Over the past two decades, transcatheter aortic valve implantation (TAVI) has revolutionized therapeutic options of AS in patients unsuitable for a surgical approach, and further has become an established choice of treatment in patients with intermediate and low surgical risk [2] [4] [5]. However, several limitations of TAVI have been recognized. Namely, the size of a patient’s native aortic annulus is assumed to have a critical impact on device success and valve performance. Previous studies investigating early generations of TAVI prostheses have shown, that large aortic annuli are associated with higher complication rates, especially moderate and severe paravalvular leakage (PVL) as predictor of 1-year mortality, but also an increased risk for vascular complications and pacemaker implantation rates [6] [7] [8] [9].

The self-expanding Evolut R (ER) valve and the balloon-expandable SAPIEN 3 (S3) valve are frequently used third-generation TAVI prostheses. Following the manufacturer’s instructions for use (IFU), both valve prostheses are restricted to a certain annulus size, being licensed for an annulus area up to 683 mm^2^ and an annulus perimeter up to 94.2 mm in case of the S3 29 mm or the ER 34 mm, respectively. Yet, patients with severe AS and an aortic annulus size exceeding the manufacturer’s recommendations might be ineligible for surgical aortic valve replacement and benefit from off-label TAVI with either of these valve prostheses. However, evidence for outcome and success rates in these patients is limited to studies with modest patient numbers [7] [10, 11]. Therefore, the aim of this study was to evaluate outcomes and valve performance of the S3 29 mm and ER 34 mm TAVI prostheses in patients with extra-large aortic annulus (ELA) exceeding the manufacturer’s sizing recommendations in comparison to patients treated within the IFU sizing range.

## Methods

### Study population

**Fig. 1:**
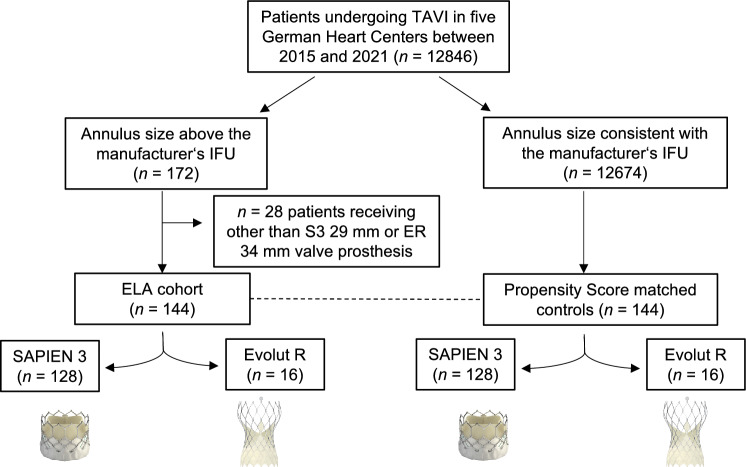
Study flow chart. Patients from 5 German Heart Centers undergoing TAVI procedure between January 2015 and December 2021 were screened for an aortic annulus size beyond the instruction for use (IFU) of the respective implanted valve. Of those, 144 patients that received either the balloon-expandable SAPIEN 3 (S3) 29 mm or the Evolut R (ER) 34 mm valve were included for further analysis. 28 patients received other than S3 29 mm or ER 34 mm valve prosthesis. Included patients were matched to controls with an annulus size within the permitted range of the manufacturer’s IFU by propensity score matching. *ELA* extra-large annulus

12,846 patients from five German high volume centers undergoing TAVI between January 2015 and December 2021 were retrospectively scrutinized for annulus size and implanted valve type. Inclusion criteria were (1) implantation of the S3 29 mm or ER 34 mm TAVI prosthesis in (2) patients with an extra-large aortic annulus defined as exceeding the size recommended by the manufacturer’s IFU (annulus area > 683 mm^2^ for S3 29 mm; annulus perimeter > 94.2 mm for ER 34 mm). 172 patients (1.3%) were identified being treated for AS with TAVI despite an aortic annulus size beyond the IFU for the respective valve implanted. Of those, 144 patients (1.1%) were treated with a S3 29 mm or an ER 34 mm valve prosthesis and were compared to 144 propensity-score matched patients with an aortic annulus size within the recommended range of S3 29 mm or ER 34 mm and being treated with either of both valve types (Fig. 1). Annular measurements were performed on preoperative MSCT images routinely acquired for procedure planning with a slice thickness of 1 mm and 40 ml of intravenously administered contrast agent. Measurements were performed in systolic reconstruction using the 3mensio software. The aortic annulus plane was defined by the nadirs of the three coronary cusps. Data collection was performed according to the requirements of the centers’ ethics committees and complied with the declaration of Helsinki. All patients gave written informed consent for the procedure.

### Outcomes

Transthoracic echocardiography (TTE) was performed before discharge to assess transvalvular gradients, degree of paravalvular regurgitation and left ventricular ejection fraction (LVEF). Paravalvular regurgitation was graded using a multiparametric and integrative approach according to current recommendations [12]. Device success was defined according to the Valve Academic Research Consortium 3 (VARC 3) definitions as the correct positioning of one single valve with absence of more than mild PVL, a post-interventional transvalvular gradient < 20 mmHg, freedom from procedure-related complications requiring surgery or intervention and 30-day survival. All further endpoints and categorization of complications are reported according to VARC-3 definitions [13].

### Statistical analysis and propensity score matching

Continuous variables are presented as mean ± standard deviation (SD), categorical variables as percentages. Statistical analysis for continuous variables was performed with student’s t-test when normally distributed, otherwise Mann–Whitney *U*-test was used for statistical testing. For categorical variables, *χ*^2^-test or Fisher’s exact test was calculated. Results were assumed significant at a two-sided alpha level < 0.05. Data analysis was carried out with GraphPad Prism v8.4.0 (GraphPad Software Inc., San Diego, CA), SPSS 27.0 for Windows (IBM Corporation, Armonk, NY) and *R* version 4.1.2 (*R* Foundation for Statistical Computing, Vienna, Austria).

Propensity score matching (PSM) was applied to adjust for differences in pre-procedural patient characteristics, comorbidities and procedure-related features of patients treated within or outside the intended sizing range of the prostheses. Propensity score was derived from a logistic regression model including the following 17 variables: sex, age, body mass index (BMI), chronic obstructive pulmonary disease (COPD), periphery artery disease, arterial hypertension, diabetes mellitus, coronary artery disease, previous cardiac surgery, pre-existing permanent pacemaker, atrial fibrillation, New York Heart Association (NYHA) functional class, glomerular filtration rate (GFR), EuroSCORE II, left ventricular ejection fraction (LVEF), valve type and vascular access. A 1:1 nearest neighbor matching algorithm without replacement and a caliper setting of 0.2 was applied. Balance between comparator baseline characteristics was defined as an absolute standardized mean difference < 20% (Suppl. Fig. S1).

## Results

### Patient characteristics and anatomical features

144 patients with ELA were propensity score matched to a control cohort of patients treated with the same valve prosthesis within the intended sizing range **(**Fig. 1**)**. Baseline patient characteristics are presented in Table 1. By PSM, relevant differences in preexisting conditions between both groups could be eliminated with respect to age, NYHA functional class, LVEF, vascular access, selected valve type and diabetes mellitus. (Suppl. Fig. S1). Patients in both groups were predominantly male (93.8%) with a mean age of 78 years. Average height and weight were significantly higher in the ELA group.

**Table 1:** Patient characteristics after propensity score matching

	ELA cohort (*n* = 144)	Control cohort (*n* = 144)	*p*-value
Male sex	135 (93.8%)	135 (93.8%)	1.00
Age (years)	77.7 ± 7.5	78.4 ± 7.3	0.42
Height (cm)	177.7 ± 7.7	175.9 ± 7.0	0.03
Weight (kg)	89.3 ± 19.7	83.2 ± 14.0	0.02
Body mass index (kg/m^2^)	28.2 ± 5.7	26.8 ± 4.2	0.14
EuroSCORE II (%)	5.4 ± 6.0	4.7 ± 4.0	0.96
STS-Score (%)	3.4 ± 2.3	3.7 ± 4.8	0.39
Chronic obstructive pulmonary disease	31 (21.5%)	31 (21.5%)	1.00
Peripheral artery disease	46 (31.9%)	43 (29.9%)	0.70
Arterial Hypertension	124 (86.1%)	124 (86.1%)	1.00
Diabetes mellitus	38 (26.4%)	37 (25.7%)	1.00
Coronary Artery Disease	83 (57.6%)	71 (49.3%)	0.19
Previous cardiac surgery	21 (14.6%)	17 (11.8%)	0.60
Previous permanent pacemaker implantation	25 (17.4%)	24 (16.7%)	1.00
Atrial Fibrillation	66 (46.2%)	71 (49.3%)	0.64
Previous stroke or transient ischemic attack	23 (16.0%)	15 (12.2%)	0.59
NYHA class III/IV	100 (69.4%)	100 (69.4%)	1.00
Creatinine	1.5 ± 1.3	1.4 ± 1.1	0.44
GFR > 60 ml/min	74 (51.4%)	77 (53.5%)	
GFR 30-60 ml/min	56 (38.9%)	55 (38.2%)	0.89
GFR < 30 ml/min	14 (9.7%)	12 (8.3%)	
Dialysis	6 (4.2%)	4 (2.8%)	0.75

Mean values ± SD or incidences with percentages are shown. *NYHA* New York Heart Association, *EuroSCORE II* European System for cardiac operative risk evaluation II, *GFR* Glomerular filtration rate, *NYHA* New York Heart Association, *STS-Score* Society of thoracic surgeons score

Per definition, anatomical features of the aortic annulus, namely annulus area, perimeter and diameter were significantly larger in the ELA group as assessed by multidetector computed tomography **(**Table 2**)**. 15 patients with an annulus area > 683 mm^2^ who received the S3 valve had a perimeter < 94.2 mm^2^. One patient with an annulus perimeter > 94.2 mm^2^ was treated with an Evolute R prosthesis despite an anulus area < 683 mm^2^. Echocardiographic characteristics including LVEF, aortic valve area, mean and peak transvalvular pressure gradients and degree of aortic regurgitation were similar in the ELA and control group, respectively.

**Table 2:** Anatomical features of the aortic valve, aortic annulus and left ventricular function assessed by multidetector computed tomography and transthoracic echocardiography

	ELA cohort	Control cohort	*p*-value
**Multidetector computed tomography**			
Annulus area (mm^2^)	730.4 ± 53.9	586.0 ± 48.2	< 0.01
Perimeter (mm)	96.7 ± 6.5	93.2 ± 67.8	< 0.01
Annulus diameter min (mm)	27.2 ± 1.7	24.3 ± 1.7	< 0.01
Annulus diameter max (mm)	33.9 ± 1.6	30.7 ± 1.5	< 0.01
Agatston-Score	1563.5 ± 1667.38	1813 ± 1814	0.70
Left coronary artery height (mm)	16.7 ± 4.0	14.8 ± 3.2	< 0.01
Right coronary artery height (mm)	19.7 ± 3.8	18.4 ± 4.0	0.01
**Transthoracic echocardiography**			
Left ventricular ejection fraction			
> 50%	64 (44.4%)	65 (45.1%)	
40–49%	29 (20.1%)	32 (22.2%)	0.88
30–39%	28 (19.4%)	23 (16.0%)	
< 30%	23 (16.0%)	24 (16.7%)	
Mean transvalvular pressure gradient (mmHg)	38,9 ± 17.9	39.0 ± 15.6	0.68
Peak transvalvular pressure gradient (mmHg)	63.6 ± 27.7	61.3 ± 23.4	0.87
Aortic valve area (cm^2^)	0.80 ± 0.2	0.75 ± 0.2	0.06
Aortic regurgitation			
None	48 (34.8%)	34 (25.4%)	
Mild	64 (46.4%)	75 (56.0%)	0.23
Moderate	20 (14.5%)	22 (16.4%)	
Severe	6 (4.4%)	3 (2.2%)	

Mean values ± SD or incidences with percentages are shown

### Procedural characteristics

128 patients (88.9%) in each group were treated with the S3 29 mm, 16 patients (11.1%) with the ER 34 mm valve prosthesis **(**Table 3**)**. TAVI procedure was performed under conscious sedation in 79.9% and 73.6%, respectively (*p* = 0.21). A transfemoral access was chosen in 88.2% and 88.9%. Procedure time was identical in the ELA and control cohort (85 ± 42 min vs. 85 ± 39 min; *p* = 0.93). Numerically, pre- and post-dilatation was performed more frequently in the ELA group compared to the control cohort without reaching statistical significance (pre-dilatation: 43.7% vs. 35.0%; *p* = 0.13; post-dilatation: 13.5% vs. 8.3%; *p* = 0.16). Selected balloon size was bigger in ELA patients than in control patients for pre- (25 ± 2.7 vs. 23 ± 3.1 mm; *p* = 0.04) and post-dilatation (28 ± 2.1 vs. 26 ± 2.3 mm; *p* = 0.09). Additional volume was added to the balloon in 10 patients of the S3 ELA group (mean additional filling volume 2.2 ± 0.8 ml) and only in one patient of the control group (2 ml additional filling volume, *p* < 0.01).

**Table 3:** Procedural features. Mean values ± SD or incidences with percentages are shown

	ELA cohort	Control cohort	*p-*value
Anesthesia			
Conscious sedation	115 (79.9%)	106 (73.6%)	
General anesthesia	29 (20.1%)	38 (26.4%)	0.21
Valve Type			
SAPIEN 3	128 (88.9%)	128 (88.9%)	
Evolut R	16 (11.1%)	16 (11.1%)	1.00
Access			
Transfemoral	127 (88.2%)	128 (88.9%)	
Others	17 (11.8%)	16 (11.1%)	0.85
Procedure Time (min)	85 ± 42	84 ± 39	0.93
Predilatation	62 (43.7%)	50 (35.0%)	0.13
Balloon size (mm)	25 ± 2.7	23 ± 3.1	0.04
Postdilatation	19 (13.5%)	12 (8.3%)	0.16
Balloon size (mm)	28 ± 2.1	25 ± 2.3	0.08
OVERSIZING	10 (6.9%)	1 (0.7%)	< 0.01
1 ml additional filling volume	1 (0.7%)	0 (0%)	
2 ml additional filling volume	7 (4.9%)	1 (0.7%)	
3 ml additional filling volume	1 (0.7%)	0 (0%)	
4 ml additional filling volume	1 (0.7%)	0 (0%)	

### Periprocedural complications and echocardiographic outcome

**Fig. 2:**
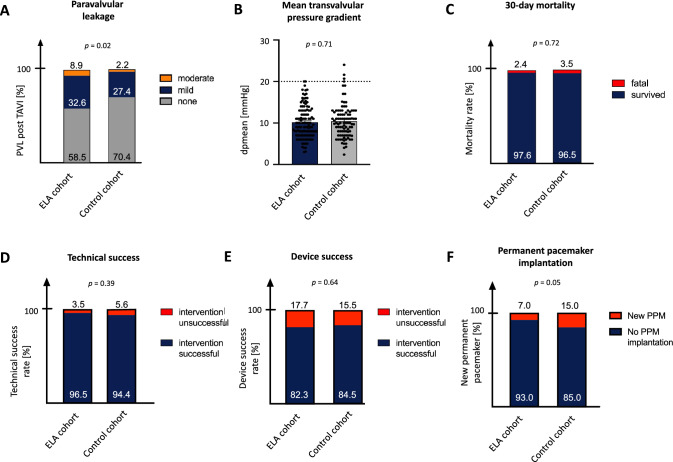
Post-procedural outcomes after TAVI with the Sapien 3 29 mm or Evolut R 34 mm prosthesis. Incidence of moderate paravalvular leakage (PVL) was significantly elevated in the extra-large annulus (ELA) cohort compared to the control group (**A**). There was no statistical difference in mean transvalvular pressure gradient (**B**), 30-day mortality (**C**) or technical success (**D**). Also, device success was comparable after TAVI (**E**). Implantation rate of new permanent pacemaker after TAVI was significantly lower in the ELA group (**F**)

Severe intraprocedural complications such as annulus rupture, cardiac tamponade or peri-interventional myocardial infarction were rare without differences between the two groups **(**Table 4**)**. Likewise, valve embolization, need for implantation of a second valve or conversion to surgery were scarce and equally distributed in both groups. Technical success defined according to VARC 3 criteria was high without significant differences between ELA patients and matched controls (96.5% vs. 94.4%; *p* = 0.39). Device success 30 days after TAVI procedure was 82.3% and 84.5% (*p* = 0.64), respectively. Pacemaker implantation rate after TAVI was lower in the ELA cohort as compared to the control group (7.0% vs. 15.0%; *p* = 0.05). Type 3 or 4 bleeding did not appear more frequently in the ELA group (3.5% vs 2.8%; *p* = 0.36). Also, major vascular complications did not vary between the investigated cohorts (3.5% vs 4.9%; *p* = 0.28). Furthermore, incidence of peri-interventional stroke (2.1% vs. 2.1%; *p* = 1.0) or acute kidney injury (13.6% vs. 12.5%; *p* = 0.79) was comparable. No significant differences in ICU (4.7 ± 11.1 d vs. 3.3 ± 6.6 d; *p* = 0.26) or total hospital stay (12.6 ± 14.4 days vs. 10.8 ± 8.7 days; *p* = 0.29) were observed in the ELA group. The incidence of PVL was higher in the ELA cohort (41.5% vs. 29.6%; *p* = 0.04) with significantly more cases of moderate PVL (8.9% vs. 2.2%; *p* = 0.02) and no cases of severe PVL in both groups (Fig. 2). Post-interventional mean transvalvular gradient was similar in both groups with 10.2 ± 3.6 mmHg vs. 10.4 ± 4.0 mmHg (*p* = 0.71), but 3 patients (2%) of the control patients presented with a mean transvalvular gradient above 20 mmHg compared to 0% in the ELA group. 30-day mortality did not differ significantly between ELA and control cohort (2.5% vs. 3.4%; *p* = 0.72). Overall, we noticed a trend towards post-interventional improvement of LVEF compared to baseline echocardiographic measurements.

**Table 4:** Complication rates and secondary outcomes. Mean values ± SD or incidences with percentages are shown

	ELA cohort	Control cohort	*p*- value
Annulus rupture	0 (0%)	0 (0%)	1.00
Cardiac tamponade	2 (1.4%)	0 (0%)	0.51
Coronary obstruction	0 (0%)	1 (0.7%)	1.00
Valve embolization	0 (0%)	0 (0%)	1.00
Need for second valve	1 (0.7%)	0 (0%)	1.00
Conversion to surgery	1 (0.7%)	1 (0.7%)	1.00
Technical success	139 (96.5%)	136 (94.4%)	0.39
Device success	102 (82.3%)	102 (84.5%)	0.64
New permanent pacemaker*	8 (7.0%)	18 (15.0%)	0.05
Bleeding			
Type 1	16 (11.1%)	10 (6.9%)	
Type 2	5 (3.5%)	2 (1.4%)	0.36
Type 3	5 (3.5%)	4 (2.8%)	
Type 4	0 (0%)	0 (0%)	
Vascular complications			
Minor	16 (11.1%)	10 (9.0%)	0.28
Major	5 (3.5%)	9 (4.9%)	
Stroke	3 (2.1%)	3 (2.1%)	1.00
Akute kidney injury	19 (13.6%)	18 (12.5%)	0.79
30-day mortality	3 (2.4%)	5 (3.5%)	0.72
Left ventricular ejection fraction			
> 50%	65 (51.2%)	56 (56.0%)	
40–49%	25 (29.7%)	20 (20.0%)	0.39
30–39%	26 (10.4%)	14 (14.0%)	
< 30%	21 (8.7%)	10 (10.0%)	
ICU stay	4.7 ± 11.1	3.3 ± 6.6	0.26
Total hospital stay	12.6 ± 14.4	10.8 ± 8.7	0.29

^*^Patients with pre-existing permanent pacemakers were excluded from analysis. *ICU* intensive care unit

### Subgroup analysis of SAPIEN 3 and Evolut R treated patients

In a subgroup analysis of 128 ELA patients treated with the S3 29 mm TAVI prosthesis, no increased risk was detected for peri-interventional annulus rupture, cardiac tamponade, coronary obstruction or valve embolization with the need for implantation of a second valve or conversion to a surgical approach as compared to matched control patients treated with the same valve type (Table 5). Implantation rate of a new permanent pacemaker was numerically diminished in ELA patients in contrast to control patients (4.8% vs. 13.5%; *p* = 0.10). Bleeding complications type 3 or 4 appeared at similar levels in both groups (2.3% vs. 3.1%; *p* = 0.58), also major vascular complications were comparable between both cohorts (2.3% vs. 6.3%; *p* = 0.22). Neither for peri-interventional stroke or acute kidney injury, nor for ICU or hospital stay duration, significant differences were detected between ELA and control patients treated with the S3 29 mm valve. In neither of both cohorts, severe PVL was noticed after TAVI, but incidence of moderate PVL was significantly elevated in the ELA group compared to matched control patients (8.3% vs. 1.7%; *p* = 0.04). Interestingly, technical (93%) and device success (81%) were numerically lowest in the upper tertile of ELA patients, however still on acceptable levels and comparable to the other tertiles. Also, there was no statistically significant difference in intraprocedural complication rates in the upper tertile of ELA-patients (Table S1). ICU and hospital stay was longer in patients treated early (01/2015—06/2018) compared to patients treated after 06/2018. Numerically, device success was lower in the early treatment group and pacemaker implantation rate, vascular complications and stroke occurred more frequently (Table S2).

**Table 5:** Subgroup analysis according to the implanted valve type

	ELA S3	Matched controls	*p*-value	ELA ER	Matched controls	*p*-value
Annulus area (mm^2^)	729.0 ±46.4	586.2 ±47.8	<0.01	721.6 ±49.4	584.9 ±52.7	<0.01
minimum / maximum	683.2 / 939.9 mm^2.^	432.1 / 677.3 mm^2^		678.4 / 850.0 mm^2^	447.4 / 670.7 mm^2^	
Annulus perimeter (mm)	96.7 ±6.9	87.2 ±3.4	<0.01	97.1 ±2.3	86.9 ±4.1	<0.01
minimum/maximum	89.9 / 110.1 mm	75.7 / 93.7 mm		94.3 / 105.0 mm	76.3 / 93.3 mm	
Annulus rupture	0 (0%)	0 (0%)	1.0	0 (0%)	0 (0%)	1.00
Cardiac tamponade	2 (1.6%)	0 (0%)	0.51	0 (0%)	0 (0%)	1.00
Coronary obstruction	0 (0%)	1 (0.8%)	1.0	0 (0%)	0 (0%)	1.00
Valve embolization	0 (0%)	0 (0%)	1.0	0 (0%)	0 (0%)	1.00
Need for second valve	1 (0.8%)	0 (0%)	1.0	0 (0%)	0 (0%)	1.00
Conversion to surgery	1 (0.8%)	1 (1.1%)	1.0	0 (0%)	0 (0%)	1.00
Technical success	125 (98.0%)	121 (94.5%)	0.20	14 (87.5%)	15 (93.8%)	0.54
Device success	95 (85.6%)	82 (84.5%)	0.83	10 (62.5%)	14 (87.5%)	0.22
New permanent pacemaker*	6 (4.8%)	14 (13.5%)	0.10	2 (14.3%)	4 (25%)	0.66
Bleeding						
Type 1	15 (11.7%)	8 (6.3%)		1 (6.3%)	2 (12.5%)	
Type 2	5 (3.9%)	2 (1.6%)	0.58	0 (0%)	0 (0%)	0.48
Type 3	3 (2.3%)	4 (3.1%)		2 (12.5%)	0 (0%)	
Type 4	0 (0%)	0 (0%)		0 (0%)	0 (0%)	
Vascular Complication						
Minor	15 (11.7%)	8 (6.3%)	0.22	1 (6.3%)	2 (12.5%)	0.54
Major	3 (2.3%)	8 (6.3%)		2 (12.5%)	1 (6.3%)	
Stroke	3 (2.4%)	3 (2.3%)	1.0	2 (13.3%)	0 (0%)	1.00
Akute kidney injury	17 (13.6%)	15 (11.7%)	0.71	2 (13.3%)	3 (18.8%)	1.00
30-day mortality	1 (0.9%)	5 (3.9%)	0.21	2 (14.3%)	0 (0%)	0.23
ICU stay	4.7 ±11.7	3,5 ±7.0	0.36	4.1 ±2.7	2.1 ±1.3	0.02
Total hospital stay	12.7 ±15.1	11.2 ±9.1	0.42	11.6 ±4.3	8.2 ±3.5	0.04
Paravalvular Leakage						
Mild	35 (29.1%)	31 (26.1%)		9 (60.0%)	6 (37.5%)	
Moderate	10 (8.3%)	2 (1.7%)	0.04	2 (13.3%)	1 (6.3%)	0.24
Severe	0 (0%)	0 (0%)		0 (0%)	0 (0%)	

Mean values ± SD or incidences with percentages are shown. ^*^Patients with pre-existing permanent pacemakers were excluded from analysis. *ICU* intensive care unit

Similar to the S3 subgroup, main intra-procedural events and complication rates were not elevated in the ELA cohort when receiving an ER valve prosthesis compared to matched controls. No differences were observed with regard to technical success (87.5% vs. 93.8%; *p* = 0.54) or device success (62.5% vs. 87.5%; *p* = 0.22), the latter of which was numerically reduced in the ELA group due to two fatal cases within 30 days after TAVI procedure. Implantation rate of a new permanent pacemaker was numerically lower in patients with ELA (14.3% vs 25.0%; *p* = 0.66). Generally, the need for implantation of a pacemaker after TAVI was higher in patients treated with the ER prosthesis than when using the S3 prosthesis. No differences were seen in bleeding or vascular complications, stroke or acute kidney injury after TAVI in ELA patients receiving the ER prosthesis compared to controls. ICU (4 ± 3 vs. 2 ± 1 days; *p* = 0.02) and hospital stay (12 ± 4 vs. 8 ± 4 days; *p* = 0.04) were longer in the ELA group and PVL was numerically elevated, yet not reaching statistical significance.

## Discussion

The present study reports outcomes of the to date largest real-world cohort of patients with ELA being treated with a third-generation TAVI prosthesis for AS despite an annulus size exceeding the permitted annulus size. Key findings of our work are: (1) TAVI in patients with ELA was feasible and safe providing comparable results as matched control patients receiving the same valve prosthesis within the intended sizing range. (2) Peri-procedural complications including stroke, major bleeding or vascular complications were similar in ELA and matched control patients, respectively. (3) The incidence of moderate PVL was higher in ELA patients, while less ELA patients were in need for a new permanent pacemaker after TAVI.

Compared to surgical aortic valve replacement, the selection of TAVI prosthesis sizes is rather limited. As a consequence, the annulus size of 1–2% of patients considered unsuitable for surgery lies outside of the approved sizing range of commonly used TAVI prostheses [9] [10] [14]. The lack of approved therapies necessitates particular attention of the treating interventional team for patients with ELA. This multi-center study proves TAVI to be a feasible and safe treatment option in patients with an annulus size up to 939.9 mm^2^. Despite the potential risks associated with the implantation of an in relation to native annulus size undersized prosthesis, technical success was similar for ELA and control patients in this study, being 96.5% and 94.4%, respectively. Likewise, device success was comparable in both groups. Previous data on this particular patient group is limited. Schaefer et al. reported the results of a small single-center case series of ELA patients treated with the S3 29 mm prosthesis, confirming safety and feasibility in ELA patients even with a device success rate of 100% [10]. Our findings are further supported by an analysis of the TAVR-Large Registry [14]. In the cohort reported here, no significant differences between ELA and control patients were observed for any adverse peri- or post-procedural event including type 3/4 bleeding or vascular complications as well as severe intra-procedural adverse events.

Similar to the study by Armijo et al., the incidence of new onset conduction disturbances requiring permanent pacemaker implantation was significantly lower with only 7% in the ELA group as compared to 15% in matched controls [14]. This observation might be explained by larger aortic annulus morphologies resulting in less oversizing and as a consequence less pressure on the cardiac conduction system after deployment of the valve prosthesis. On the other hand, as trade off this mechanism may contribute to a higher risk of PVL. In line with that, ELA patients had a fourfold increased risk for moderate PVL compared to matched controls in our study as has been observed previously [6] [7] [9]. Conclusively, patients with ELA in our study were taller and heavier, native aortic valve area was significantly larger despite similar transvalvular gradients, and oversizing as well as intra-procedural pre- or post-dilatation was applied more frequently using bigger balloons than in the matched control cohort.

In a recent report from the German Aortic Valve Registry on patients with large and extra-large aortic annuli receiving either the first-generation Edwards SAPIEN or the Medtronic CoreValve, the authors describe an increasing utilization of TAVI in patients with large and extra-large aortic annuli from 2011 to 2017, highlighting the growing operator’s experience and confidence in treating patients outside the approved sizing recommendation [9]. For first-generation TAVI prostheses, Piayda et al. observed an elevated risk of moderate and severe PVL and major vascular complications in patients with ELA as compared to large annulus controls. Additionally, pacemaker implantation rate was significantly higher after TAVI in the ELA cohort [9]. In our patient collective treated with a third-generation TAVI prosthesis, we could observe an improvement with regard to these endpoints: although PVL rate was still higher in the ELA cohort, vascular complications were comparable in both groups and pacemaker implantation rate was even lower in ELA patients. Hence, increasing experience, technological progress and optimization of valve prostheses have contributed improving the outcome of TAVI in patients with difficult annular anatomies. Nonetheless, PVL remains an issue of concern in patients with ELA, even when treated with a third-generation prosthesis. The manufacturing of larger prostheses with more oversizing may address this clinical need and particular attention should be paid to patients with ELA by the interventional team. In this context, new TAVI prostheses have been developed recently, addressing the problem of extra-large aortic annuli and allowing for usage in annulus anatomies with an annulus area of up to 840 mm^2^ and a perimeter of 100.5 mm [15]. However, large-scale studies and investigations on long-term outcomes of these valves are still missing.

Although we did not observe an increased short-term mortality risk, several studies report a correlation of moderate or even mild PVL with worse long-term outcome after TAVI [16] [17] [18]. Follow up studies on patients with ELA are necessary to evaluate survival rate and outcome after a longer observational period.

In a comparison of S3 and ER in an unselected collective including patients with both large and extra-large annuli, Armijo et al. report a superior performance of the S3 prosthesis with significantly lower rates of valve embolization and need for implantation of a second valve, less moderate or severe PVL, lower device failure rates and all-cause mortality [14]. On the other hand, in a subgroup analysis including only ELA patients, no significant differences were detected between S3 and ER prosthesis. In our study, PVL was significantly increased in S3, but not in the ER subgroup, whereas ICU and hospital stay were significantly elevated only in the ER cohort. Numerically, bleeding or vascular complications, mortality and device failure rates were more frequent in the ER subgroup. Although representing the largest ELA cohort treated with third-generation TAVI prostheses, the distribution of self-expanding and balloon-expandable valve prostheses was uneven within our patient collective and predominantly S3 prostheses were used, thus limiting the conclusions regarding the use of ER. Consequently, the comparative subgroup analyses on S3 and ER should be considered merely descriptive since the small number of included ER patients precludes any statistically solid conclusion.

## Conclusion

Catheter based aortic valve replacement with the S3 29 mm and the ER 34 mm is feasible and safe in patients with ELA and provides acceptable outcomes and complication rates, comparable to results in patients with normal-sized aortic annuli. Pacemaker implantation rate is lower in ELA patients, yet the incidence of post-interventional PVL is elevated and remains an issue that needs to be addressed by manufacturers and operators.

### Supplementary Information

Below is the link to the electronic supplementary material.

Supplementary file1 (DOCX 450 KB)

## Data Availability

Data are available on reasonable request.

## Abbreviations

AS: Aortic valve stenosis
ELA: Extra-large annulus
ER: Evolut R
IFU: Instructions for use
NYHA: New York Heart Association
PSM: Propensity score matching
PVL: Paravalvular leakage
S3: SAPIEN 3
TAVI: Transcatheter aortic valve implantation
VARC 3: Valve Academic Research Consortium 3
