# Supplementary material for: Feasibility and outcome of third-generation transcatheter aortic valve implantation in patients with extra-large aortic annulus
Source: Clin Res Cardiol. 2023 Jul 31;113(1):107–15. doi: 10.1007/s00392-023-02278-1 (PMC10808444; doi:10.1007/s00392-023-02278-1)
Supplement: Supplementary file 1 — Supplementary file1 (DOCX 450 KB) [file 392_2023_2278_MOESM1_ESM.docx]

Supplementary data

**Supplementary figure S1:** Results of propensity-score matching based on 17 matching criteria. Patient characteristics and procedural features were considered. Standardized mean difference in all matching criteria are shown before and after propensity-score matching. NYHA: New York Heart Association. EuroSCORE II: European System for cardiac operative risk evaluation II.

|  | **1^st^ tertile (*n* = 42)** |  | **2^nd^ tertile (*n* = 43)** | **3^nd^ tertile (*n* = 43)** | ***p*- value** |
| --- | --- | --- | --- | --- | --- |
| ***Annulus area (mm^2^)*** | 690.1 ±5.0 |  | 717.0 ±10.6 | 780.1 ± 45.8 | **<0.01** |
| ***Annulus perimeter (mm)*** | 94.4 ±1.4 |  | 96.5 ±1.2 | 99.1 ±11.2 | **<0.01** |
| ***Annulus rupture*** | 0 (0%) |  | 0 (0%) | 0 (0%) | **1.00** |
| ***Cardiac tamponade*** | 1 (2.3%) |  | 0 (0%) | 1 (2.3%) | **1.00** |
| ***Coronary obstruction*** | 0 (0%) |  | 0 (0%) | 0 (0%) | **1.00** |
| ***Valve embolization*** | 0 (0%) |  | 0 (0%) | 0 (0%) | **1.00** |
| ***Need for second valve*** | 0 (0%) |  | 0 (0%) | 1 (2.3%) | **1.0** |
| ***Conversion to surgery*** | 0 (0%) |  | 0 (0%) | 1 (2.3%) | **0.34** |
| ***Technical success*** | 42 (100.0%) |  | 43 (100%) | 40 (93.0%) | **0.24** |
| ***Device success*** | 31 (86.1%) |  | 34 (89.5%) | 30 (81.0%) | **0.19** |
| ***New permanent pacemaker**** | 1 (2.3%) |  | 2 (4.7%) | 3 (7.0 %) | **0.40** |
| ***Bleeding*** |  |  |  |  |  |
| *type 1* | 6 (14.3%) |  | 1 (2.3%) | 8 (18.6%) |  |
| *type 2* | 3 (7.1%) |  | 1 (2.3%) | 1 (2.3%) | **0.59** |
| *type 3* | 0 (0%) |  | 1 (2.3%) | 2 (4.7%) |  |
| *type 4* | 0 (0%) |  | 0 (0%) | 0 (0%) |  |
| ***Vascular complications*** |  |  |  |  |  |
| *minor* | 7 (16.7%) |  | 2 (4.7%) | 6 (14.0%) | **0.57** |
| *major* | 1 (2.3%) |  | 1 (2.3%) | 1 (2.3%) |  |
| ***Stroke*** | 2 (4.8%) |  | 1 (2.3%) | 0 (0%) | **0.55** |
| ***Akute kidney injury*** | 6 (14.3%) |  | 9 (21.0%) | 2 (4.7%) | **0.08** |
| ***30-day mortality*** | 0 (0.0%) |  | 1 (2.3%) | 0 (0.0%) | **1.0** |
| ***ICU stay*** | 3.36 ±3.36 |  | 4.4 ±8.5 | 3.6 ±3.6 | **0.51** |
| ***Total hospital stay*** | 12.0 ±10.5 |  | 10.6 ±8.4 | 12.1 ±7.9 | **0.34** |

**Supplementary Table S1**

**Supplementary table S1:** ELA patients from the S3 cohort were divided into tertiles according the annulus area and outcomes were compared. ^*^Patients with pre-existing permanent pacemakers were excluded from analysis. ICU: intensive care unit.

**Supplementary Table S2**

|  | **01/2015-06/18** | **07/18-12/21** | ***p*- value** |
| --- | --- | --- | --- |
| ***Annulus area (mm^2^)*** | 733.4 ±52.7 | 725.9 ±39.2 | **0.30** |
| ***Annulus perimeter (mm)*** | 96.6 ±9.3 | 97.0 ±2.6 | **0.84** |
| ***Annulus rupture*** | 0 (0%) | 0 (0%) | **1.00** |
| ***Cardiac tamponade*** | 1 (1.6%) | 1 (1.6%) | **1.00** |
| ***Coronary obstruction*** | 0 (0%) | 0 (0%) | **1.00** |
| ***Valve embolization*** | 0 (0%) | 0 (0%) | **1.00** |
| ***Need for second valve*** | 1 (1.6%) | 0 (0%) | **1.00** |
| ***Conversion to surgery*** | 0 (0%) | 1 (1.6%) | **1.00** |
| ***Technical success*** | 63 (98.4%) | 62 (96.9%) | **1.0** |
| ***Device success*** | 43 (82.7%) | 52 (88.1%) | **0.43** |
| ***New permanent pacemaker**** | 5 (9.1%) | 1 (2.0%) | **0.21** |
| ***Bleeding*** |  |  |  |
| *type 1* | 10 (15.6%) | 5 (7.8%) |  |
| *type 2* | 4 (6.3%) | 1 (1.6%) | **0.72** |
| *type 3* | 1 (1.6%) | 2 (3.1%) |  |
| *type 4* | 0 (0%) | 0 (0%) |  |
| ***Vascular complications*** |  |  |  |
| *minor* | 9 (14.1%) | 6 (9.4%) | **0.61** |
| *major* | 2 (3.1%) | 1 (1.6%) |  |
| ***Stroke*** | 3 (4.9%) | 0 (0%) | **0.24** |
| ***Akute kidney injury*** | 8 (12.5%) | 9 (14.1%) | **1.00** |
| ***30-day mortality*** | 1 (1.6%) | 0 (0%) | **1.00** |
| ***ICU stay*** | 5.0 ±7.4 | 2.5 ±2.4 | **0.01** |
| ***Total hospital stay*** | 14.0 ±11.3 | 9.0 ±4.3 | **<0.01** |

**Supplementary table S2:** ELA patients from the S3 cohort were divided according to timepoint of intervention into an early (01/2015-06/2018) and a late intervention cohort (07/2018-12/2021) and outcomes were compared. ^*^Patients with pre-existing permanent pacemakers were excluded from analysis. ICU: intensive care unit.
